# Quality of Web Information About Palliative Care on Websites from the United States and Japan: Comparative Evaluation Study

**DOI:** 10.2196/ijmr.9574

**Published:** 2018-04-03

**Authors:** Kouichi Tanabe, Kaho Fujiwara, Hana Ogura, Hatsuna Yasuda, Nobuyuki Goto, Fumiko Ohtsu

**Affiliations:** ^1^ Drug Informatics Faculty of Pharmacy Meijo University Nagoya Japan; ^2^ Modern Languages Southampton University Kanagawa Japan; ^3^ Department of Pharmacy University of Fukui Hospital Fukui Japan

**Keywords:** internet, website, reliability, quality, usefulness, palliative care, consumer health information, evaluation studies

## Abstract

**Background:**

Patients and their families are able to obtain information about palliative care from websites easily nowadays. However, there are concerns on the accuracy of information on the Web and how up to date it is.

**Objective:**

The objective of this study was to elucidate problematic points of medical information about palliative care obtained from websites, and to compare the quality of the information between Japanese and US websites.

**Methods:**

We searched Google Japan and Google USA for websites relating to palliative care. We then evaluated the top 50 websites from each search using the DISCERN and LIDA instruments.

**Results:**

We found that Japanese websites were given a lower evaluation of reliability than US websites. In 3 LIDA instrument subcategories—engagability (*P*<.001), currency (*P*=.001), and content production procedure (*P*<.001)—US websites scored significantly higher and had large effect sizes.

**Conclusions:**

Our results suggest that Japanese websites have problems with the frequency with which they are updated, their update procedures and policies, and the scrutiny process the evidence must undergo. Additionally, there was a weak association between search ranking and reliability, and simultaneously we found that reliability could not be assessed by search ranking alone.

## Introduction

The World Health Organization defines palliative care as “an approach that improves the quality of life of patients and their families facing the problems associated with life-threatening illness, through the prevention and relief of suffering by means of early identification and impeccable assessment and treatment of pain and other problems, physical, psychosocial and spiritual” [1]. The incorporation of palliative care into cancer treatment from the early stages also leads to the necessity of enhancing patients’ and their families’ understanding of such care [2].

On the other hand, with the spread of the internet, it has become possible for patients and their families to easily collect treatment-related information through websites. The merit of enabling users to immediately obtain extensive information has made the internet an important means to collect medical information [3]. According to the National Telecommunication Survey 2015 in Japan, internet use through mobile phones and tablet terminals is increasing in all age groups, and the increase in the rate of such use is particularly marked among those aged 60 to 79 years [4]. The rise in the number of internet users suggests that an increasing number of patients are collecting medical information using this method.

However, information available on the internet also has drawbacks, such as not necessarily being understandable for patients without expertise and having low updating frequencies [5,6]. Furthermore, it has been reported that approximately 30% of those who browse webpages containing information regarding palliative care are unsatisfied due to insufficient information and detailed contents, indicating the necessity of improving the quality of such information as a challenge [2,7].

Therefore, in this study we focused on palliative care–related information available through websites and compared the quality of the information between Japan and the United States in order to identify problems related to medical information available through websites on the internet.

## Methods

### Website Selection

**Figure 1:**
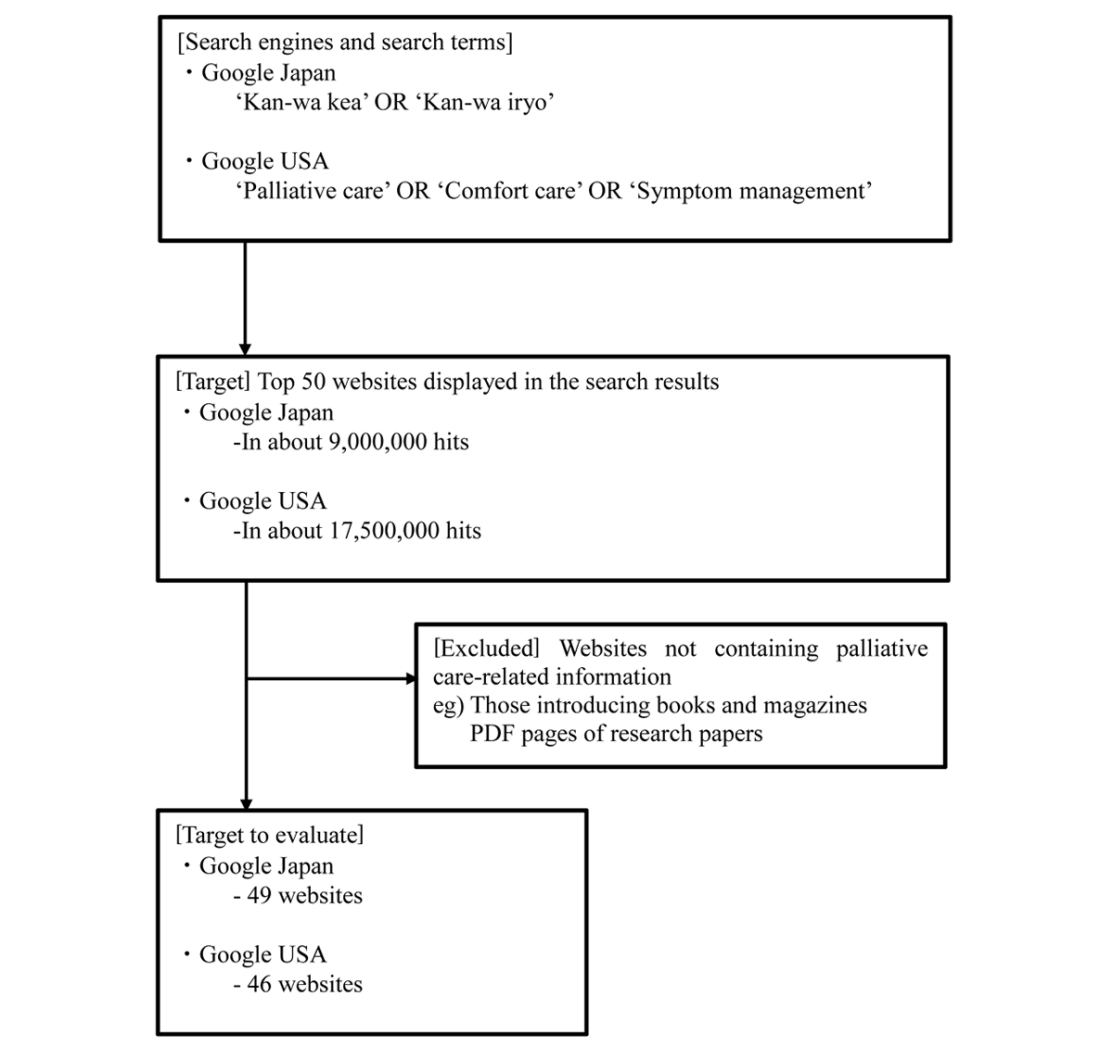
The process of selecting target websites.

Figure 1 outlines the process of selecting target websites. We searched for websites using 2 search engines: Google Japan and Google USA (Google LLC). When conducting Web searches, we turned off both the positional information and history information reference functions of the personal computer. The search terms were (palliative care OR palliative medicine [in Japanese]) on Google Japan and (palliative care OR comfort care OR symptom management) on Google USA. We targeted the top 50 websites displayed in the search results. When selecting sites, we regarded webpages having the same domain as belonging to the same website. We excluded websites not containing palliative care–related information, such as those introducing books and magazines, and with PDF pages of research papers. We ascertained that the top 50 websites found on Google USA were US sites.

### Evaluation

We evaluated the information available on the target websites by examining the owner information included in each website and by using 2 evaluation indices: the DISCERN (Textbox 1) and LIDA instruments (Textbox 2).

The DISCERN instrument [8] is an index to evaluate the quality of information regarding treatment choices, developed by Shepperd and colleagues at Oxford University, Oxford, UK. We evaluated the reliability of information (reliability-1) and the quality of information regarding treatment choices (information quality) on a 5-point scale from 1 (no) to 5 (yes). Subsequently, we evaluated overall aspects on a 5-point scale from 1 (serious or extensive shortcomings) to 5 (minimal shortcomings).

The LIDA instrument is an index to evaluate health-related information available on websites, created by Minervation, a health care consultancy [9]. It consists of 3 domains: accessibility, usefulness, and reliability (reliability-2). In this study, we rated the evaluation items on a 4-point scale from 0 (never) to 3 (always). Among the 3 domains, we evaluated only usefulness and reliability-2, as accessibility is not closely associated with reliability.

The evaluation was conducted by multiple raters independently. When interrater agreement was poor, we discussed the results with a new supervisor and used the agreed-upon results.

We divided owner information into 2 categories for evaluation: personal and organizational. We further divided organizational into medical, administrative (public), general (profit), and general (nonprofit) for comparison between Japan and the United States. We defined each category as outlined in Textbox 3.

We divided the categories of the website contents into dedicated palliative care sites, general information sites about cancer (including palliative care–related information), general medical sites (including palliative care–related information), and nonmedical sites (including palliative care–related information), and we assessed those. Textbox 4 outlines the definitions of the website content categories.

To evaluate their reliability, we also divided the target websites into 2 groups—high rank and low rank—based on their ranks when displayed in the search results. We then compared their scores for each item of the 2 evaluation indices. With regard to the reliability-related items (reliability-1 and reliability-2) of both evaluation indices, we examined the correlation between the scores for these items and ranks.

**DISCERN instrument for judging the quality of written consumer health information. Section and questions are presented below. Scored as 1=no, 3=partially, 5=yes.**

**Reliability-1: Is the publication reliable?**

- Are the aims clear?
- Does it achieve its aims?
- Is it relevant?
- Is it clear what sources of information were used to compile the publication (other than the author or producer)?
- Is it clear when the information used or reported in the publication was produced?

**Information quality: How good is the quality of information treatment choices?**

- Is it balanced and unbiased?
- Does it provide details of additional sources of support and information?
- Does it refer to areas of uncertainty?
- Does it describe how each treatment works?
- Does it describe the benefits of each treatment?
- Does it describe the risks of each treatment?
- Does it describe what would happen if no treatment is used?
- Does it describe how the treatment choices affect overall quality of life?
- Is it clear that there may be more than one possible treatment choice?
- Does it provide support for shared decision making?

**Overall evaluation: Overall rating of the publication**

- Based on the answers to all of the above questions, rate the overall quality of the publication as a source of information about treatment choices

**LIDA instrument for validation of health care websites. Level and questions are presented below. Scored from 0=never to 3=always.**

**Usefulness/usability**

*Clarity*

- Is there a clear statement of who this website is for?
- Is the level of detail appropriate to their level of knowledge?
- Is the layout of the main block of information clear and readable?
- Is the navigation clear and well structured?
- Can you always tell your current location in the site?
- Is the color scheme appropriate and engaging?

*Consistency*

- Is the same page layout used throughout the site?
- Do navigational links have a consistent function?
- Is the site structure (categories or organization of pages) applied consistently?

*Functionality*

- Does the site provide an effective search facility?
- Does the site provide effective browsing facilities?
- Does the design minimize the cognitive overhead of using the site?
- Does the site support the normal browser navigational tools?
- Can you use the site without third-party plug-ins?

*Engagability*

- Can the user make an effective judgment of whether the site applies to them?
- Is the website interactive?
- Can the user personalize their experience of using the site?
- Does the website integrate nontextual media?

**Reliability-2**

*Currency*

- Does the site respond to recent events?
- Can users submit comments on specific content?
- Is site content updated at an appropriate interval?

*Conflicts of interest*

- Is it clear who runs the site?
- Is it clear who pays for the site?
- Is there a declaration of the objectives of the people who run the site?

*Content production*

- Does the site report a clear content production method?
- Is this a robust method?
- Can the information be checked from original sources?

*Content production procedure*

- Are the audience needs identified in advance?
- Is comprehensive literature searching conducted?
- Are retrieved documents critically appraised?
- Is content authored by subject experts?
- Is content reviewed by an independent expert or panel?

*Output of content*

- Has literature searching found the right information?
- Does the content check out?
- Is the content accurate?

**Definitions of owner information categories.**

- Personal: personal websites
- Organizational: websites of organizations, such as companies and facilities
- Medical: websites of medical institutions or corporations, such as hospitals and care facilities
- General (profit): websites of for-profit organizations, such as joint-stock companies, excluding medical institutions
- General (nonprofit): websites of nonprofit or nongovernmental organizations, excluding medical institutions
- Administrative (public): websites of prefectures, states, ministries, and agencies
- Academic: websites of academic societies

**Definitions of website content categories.**

- Dedicated palliative care sites: websites from medical conferences that are dedicated to palliative care, educational and informational sites about palliative care, hospice service program informational sites, and academic journal sites about palliative care
- General information sites about cancer: educational and informational sites about cancer and informational sites about hospitals specializing in cancer treatment
- General medical sites: informational sites about home visits and hospitals
- Nonmedical sites: featured articles from newspapers, nonmedical informational sites, and online dictionaries

### Analysis

We conducted a chi-square test to compare owner information. For comparisons of reliability, quality, and usefulness of the information available on the target websites between Japan and the United States, we calculated the mean scores for the 2 evaluation indices. The *t* test was conducted (with the significance level set at .05%, and using Cohen *d* for effect size calculation) using the statistical software IBM SPSS Statistics 24 (IBM Corporation). We applied Cohen’s standard setting to measure effect size, where no effect size is 0≦ *d* ＜0.2, small effect size is 0.2≦ *d* ＜0.4, medium effect size is 0.4≦ *d* ＜0.6, and large effect size is 0.6≦ *d.* Furthermore, we examined the correlation between each score and ranks by calculating the Pearson product-moment correlation coefficient and conducting a test to confirm the absence of a correlation.

## Results

### Overview

We identified approximately 9,000,000 websites using Google Japan (date of search: December 13, 2016) and 17,500,000 websites using Google USA (December 14, 2016). After excluding 1 website from the Google Japan search and 4 from the Google USA search, we targeted 49 Japanese sites and 46 US sites for evaluation.

### Owner Information

None of the targeted websites from either Japan or the United States were personal websites. We categorized organizational websites as follows: (1) from Japan, medical: 33; general (profit): 7; general (nonprofit): 3; administrative (public): 4; and academic: 2; and (2) from the United States, medical: 24; general (profit): 7; general (nonprofit): 4; administrative (public): 7; and academic: 4. In both cases, medical websites were the most frequent (Table 1). The chi-square test revealed no significant differences in the categorization of owner information between the Japanese and US websites (*P*=.55).

### Website Content Categories

Most dedicated palliative care sites from the United States, but none of those from Japan, were hospice service program websites. Furthermore, most general medical sites from the United States were informational sites about home visits, while most of those from Japan were general hospital introductory sites.

### Evaluation Index Scores

On comparing evaluation index scores, US websites had significantly higher scores related to reliability-1 and reliability-2 than did the Japanese websites (Table 2). Scores related to information quality and usefulness did not differ markedly.

**Table 1:** Comparison of the frequency of websites with owner information and content categories between Japan and the United States.

Information categories		Japan (n)	United States (n)	*P* value^a^
**Owner information**				.55
	Medical	33	24	
	General (profit)	7	7	
	General (nonprofit)	3	4	
	Administrative (public)	4	7	
	Academic	2	4	
	Personal	0	0	
**Contents**				.005
	Palliative care related	5	20	
	General information about cancer	6	6	
	General medical	32	19	
	Nonmedical	6	1	

^a^Chi-square test.

**Table 2:** Comparison of mean evaluation index scores between Japanese and US websites.

Instruments and items			Japan (n=49)		United States (n=46)		*P* value^a^		Effect size	
**DISCERN score, mean (SD)**										
	Reliability-1			12.1 (4.6)		14.5 (5.2)		.02		0.51
	Information quality			18.6 (6.7)		19.9 (6.6)		.26		0.24
	Reliability-1 plus information quality			30.0 (11.4)		34.4 (10.6)		.07		0.43
	Overall evaluation			2.1 (0.9)		2.6 (0.9)		.007		0.59
**LIDA score, mean (SD)**										
	**Usefulness**			44.1 (7.0)		44.5 (5.4)		.61		0.26
		Clarity		14.2 (3.7)		13.6 (2.7)		.50		0.16
		Consistency		9.4 (1.6)		8.9 (0.3)		.30		0.18
		Functionality		12.5 (2.2)		12.4 (1.9)		.26		0.15
		Engagability		7.9 (1.7)		9.5 (1.8)		<.001		0.95
	**Reliability-2**			16.1 (6.3)		21.1 (8.9)		.004		0.60
		Currency		2.8 (1.8)		3.9 (1.5)		.001		0.68
		Conflicts of interest		5.0 (1.6)		5.4 (1.4)		.23		0.24
		Content production		1.6 (1.5)		1.9 (2.8)		.54		0.28
		Content production procedure		1.9 (1.9)		4.1 (3.7)		<.001		0.75
		Output of content		4.8 (1.5)		5.8 (2.0)		.009		0.54
	Usefulness plus reliability-2			60.2 (12.1)		65.3 (11.2)		.045		0.42

^a^Student *t* test.

### Reliability of the Target Websites

#### Comparison Between High- and Low-Rank Websites

The reliability of high- and low-rank websites significantly varied in both Japan and the United States. In US websites showing larger effect sizes, the difference was more marked between low-rank websites (Table 3).

Similarly, information quality, but not usefulness, also significantly varied between high- and low-rank websites from both Japan and the United States.

A large number of US websites had marks indicating certification of compliance guarantees by third parties, represented by the Health on the Net (HON) Foundation. In contrast, none of the Japanese websites had such marks.

**Table 3:** Comparison of scores between high- and low-rank websites.

Instruments and items		Japan					United States			
		High (n=24)	Low (n=25)	*P* value^a^	Effect size	High (n=23)		Low (n=23)	*P* value^a^	Effect size
**DISCERN score, mean (SD)**										
	Reliability-1	14.4 (5.0)	9.9 (2.9)	<.001	1.12	17.6 (3.4)		11.3 (4.9)	<.001	1.41
	Information quality	21.8 (7.8)	15.4 (3.3)	<.001	1.06	24.3 (5.7)		15.3 (3.7)	<.001	1.83
	Reliability-1 plus information quality	34.8 (14.0)	25.3 (4.8)	.004	0.90	41.9 (7.5)		26.6 (7.3)	<.001	1.96
	Overall evaluation	2.5 (0.9)	1.6 (0.7)	<.001	1.14	3.1 (0.7)		2.0 (0.8)	<.001	1.47
**LIDA score, mean (SD)**										
	Usefulness	44.6 (5.3)	45.3 (2.1)	.64	0.16	44.0 (5.7)		44.3 (5.7)	.56	0.17
	Reliability-2	20.0 (6.9)	13.0 (2.8)	<.001	1.35	26.1 (9.0)		15.9 (9.0)	<.001	1.32
	Usefulness plus reliability-2	62.0 (16.7)	58.3 (3.8)	.28	0.32	70.2 (11.7)		60.2 (11.7)	.02	0.66

^a^Student *t* test.

**Figure 2:**
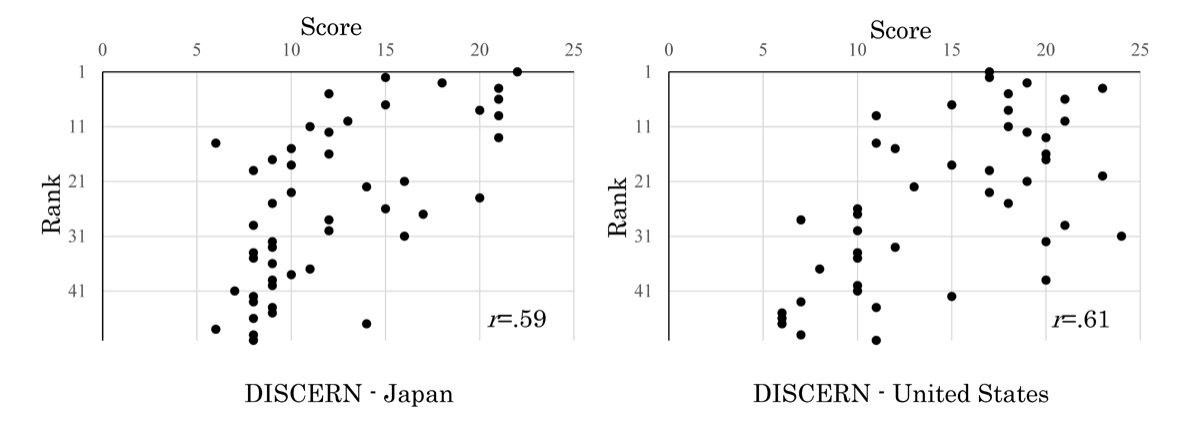
Correlation of DISCERN scores related to reliability-1 and ranks between Japanese and US websites.

**Figure 3:**
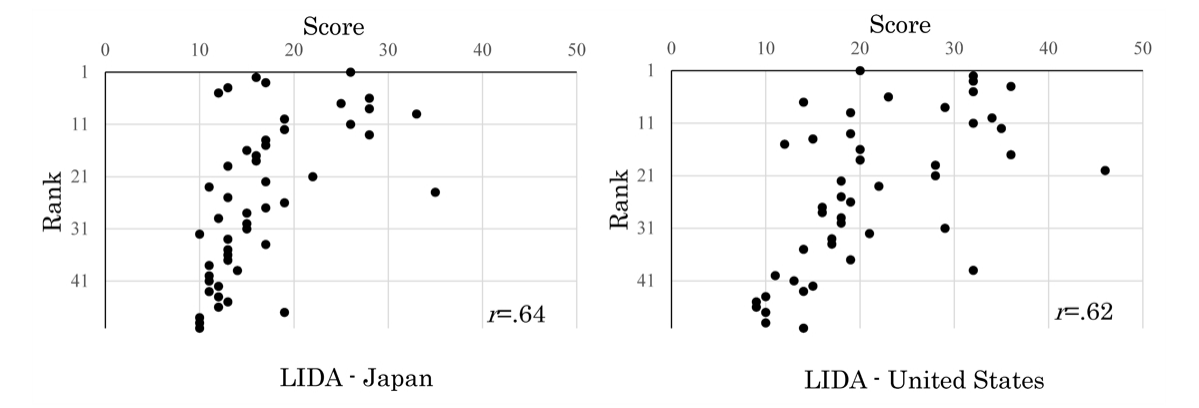
Correlation of LIDA scores related to reliability-2 and ranks between Japanese and US websites.

#### Correlation Between Reliability-Related Scores and Ranks

DISCERN (Figure 2) and LIDA (Figure 3) reliability scores of the target websites were weakly correlated with their ranks.

## Discussion

### Principal Findings

This study compared and evaluated the reliability, quality, and usefulness of palliative care information available on websites from Japan and the United States. Although the usefulness was similar between the 2 countries, the reliability and quality were lower in Japanese websites, revealing insufficient measures to provide such information with sufficient reliability in Japan. In previous studies, websites not specifying information sources and those showing profit-seeking behavior were noted as problematic [10,11]. Similarly, in this study, information sources and dates of publication were not appropriately described on some Japanese websites, revealing their insufficient usability for patients and their families. It has been suggested that Japanese palliative care sites have problems with their update frequency, their update policies and procedures, and the scrutiny process that evidence must undergo, since the scores for the US websites showed a significantly large effect size in 2 categories: currency and content production procedures, especially in the LIDA subcategory. There was no significant difference in the overall usefulness of the websites, but in the LIDA subcategory engagability, US websites had a higher score and were slightly more user friendly because many sites had interactive content and pictograms as an alternative to text. The subcategory engagability includes queries about website integration with nonverbal expression and, in the realm of drug information, the usefulness of pictograms as a tool for information communication has been reported [12,13]. This suggests that using pictograms could enhance a website’s user friendliness for users whose mother tongue is different from the language of the website. Additionally, we do not know whether any Japanese palliative care–related websites have applied the HON Foundation code of conduct (HONcode), while many English-language websites do [14-16]. A certification system such as HONcode could be useful to help determine the reliability of a website because it would be possible to refine one’s search by whether sites are certified.

Owner information categorization did not significantly vary between the 2 countries, indicating that websites belonging to medical institutions tended to be the most frequent information sources for patients in both countries. However, regarding each content category, dedicated palliative care sites ranked highest in the United States, and general medical information sites ranked highest in Japan (especially in cases where palliative care information was included in the hospital informational sites). Therefore, it may be necessary to create official sites that are dedicated to palliative care service and to provide information that is regularly reviewed and updated. Additionally, proactively applying a third-party certification system such as the HONcode is desirable because even medical experts often vary in their subjective assessments [17].

The significant difference in the level of reliability between high- and low-rank websites in both countries and the weak correlation between such levels and ranks suggest an association between the rank of each website and its reliability. The order in which websites are displayed in Google search results is based on an algorithm called PageRank [18]. This algorithm calculates original scores for individual websites based on keyword matching, the number of backlinks, updating frequency, information volume and consistency, browsing frequency, and coding appropriateness. PageRank determines where a website ranks in the search results. Therefore, the rank of a website in the search results does not necessarily reflect its reliability. However, the possibility of a website appearing high in the order as a result of being regarded as highly reliable by users and achieving a large number of accesses should also be considered. In this respect, detailed search orders themselves may not accurately reflect the reliability of websites, although those providing more reliable information tend to appear higher in the search results. In fact, in previous studies, search orders were reported to be inappropriate as a measure to examine the reliability of websites [19], and we obtained similar findings in this study. Thus, when using internet search services, the following points listed by some researchers should be noted: information contained in webpages may be inaccurate; such information is based on providers’ intentions; and websites are not primary information sources [20]. Our study also suggested the necessity of exercising caution when using information available on the internet.

With the revision of Google’ search logic, the individualization of information based on locality and access history has been promoted, but reliability-related issues have yet to be resolved. At this point, information available on websites visited by patients has been reported to be less accurate than that provided through websites targeting medical professionals [21,22]. As it may be difficult for patients without expertise to judge whether such information is sufficiently reliable, medical professionals should support patients in obtaining information that they need.

### Study Limitations

This study had some considerable limitations. First, our searches may have missed some websites. Second, we might have obtained different results using other search engines. However, although search results can differ by adding keywords, we think that this difference would be negligible and unlikely to have a large influence on the results. Third, the results might not be applicable to palliative care websites from countries other than the United States and Japan. However, although there are some limitations to this study, few studies have assessed the reliability of website information in the palliative care realm. Therefore, our results can be considered to offer future possibilities for providing information.

### Conclusions

While usefulness-related scores did not significantly vary, reliability-related scores were lower for Japanese websites. In 3 LIDA instrument subcategories, US scores were higher and had large effect sizes: engagability, currency, and content production procedure. This suggests that Japanese websites have problems in their frequency of being updated, update procedure and policy, and scrutiny process for evidence. We also clarified that the reliability of websites is weakly correlated with their ranks, but such ranks are not sufficient to judge whether the websites are sufficiently reliable. Based on these results, it may be necessary to evaluate information sources used by patients.

## Abbreviations

HON: Health on the Net
HONcode: HON Foundation code of conduct

## References

[1] World Health Organization (2018). WHO definition of palliative care.

[2] Takayasu M, Yonahara N, Matsumoto M, Sakurada T, Kobayashi E, Satoh N (2016). Research on palliative care information provision. Regul Sci Med Prod.

[3] Cullen RJ (2002). In search of evidence: family practitioners' use of the Internet for clinical information. J Med Libr Assoc.

[4] Ministry of Public Management, Home Affairs, Posts, and Telecommunications (2009). Results of the National Telecommunication Survey 2015.

[5] Watanabe K (2013). [Cancer information service for improving palliative care]. Gan Kanja To Taishoryoho.

[6] Yamada Y, Hirakata M, Todoroki K, Okazaki S, Ishiguro R, Nobutou A, Matsubara M, Kosaka M, Hata K, Iwamitsu Y (2013). The level of the information and understanding state of patients and family members before and after palliative care unit admissionvaluation of the nurses. Palliat Care Res.

[7] Pithon MM, dos Santos ES (2014). Information available on the internet about pain after orthognathic surgery: a careful review. Dental Press J Orthod.

[8] Charnock D, Shepperd S, Needham G, Gann R (1999). DISCERN: an instrument for judging the quality of written consumer health information on treatment choices. J Epidemiol Community Health.

[9] Minervation (2012). LIDA.

[10] Küçükdurmaz F, Gomez MM, Secrist E, Parvizi J (2015). Reliability, readability and quality of online information about femoracetabular impingement. Arch Bone Jt Surg.

[11] Tieman J (2016). Ensuring quality in online palliative care resources. Cancers (Basel).

[12] Liu Y, Chiu S, Lin Y, Chiou W (2014). Pictogram-based method of visualizing dietary intake. Methods Inf Med.

[13] Imanishi T, Takamatsu C, Takayama A (2017). Creation of pictograms regarding pharmacological effects of medicine: the necessity and evaluation of created pictograms. Jpn J Pharm Health Care Sci.

[14] Boyer C, Selby M, Scherrer JR, Appel RD (1998). The Health On the Net Code of Conduct for medical and health websites. Comput Biol Med.

[15] Brulet A, Llorca G, Letrilliart L (2015). Medical wikis dedicated to clinical practice: a systematic review. J Med Internet Res.

[16] Boyer C, Gaudinat A, Hanbury A, Appel RD, Ball MJ, Carpentier M, van Bemmel JH, Bergmans J, Hochstrasser D, Lindberg D, Miller R, Peterschmitt J, Safran C, Thonnet M, Geissbühler A (2017). Accessing reliable health information on the Web: a review of the HON approach. Stud Health Technol Inform.

[17] Craigie M, Loader B, Burrows R, Muncer S (2002). Reliability of health information on the Internet: an examination of experts' ratings. J Med Internet Res.

[18] Wikipedia: Google.

[19] Kawazoe S, Tsutsui A, Kishimoto K, Fukushima N (2013). A study of the health food information on Japanese websites. Shokumotu Gakkai Shi.

[20] Tanimizu M, Shinkai S, Hyodo I, Masumoto S, Nasu J, Hirazaki T (2005). Search of the Internet cancer information in the patient care. Chiro.

[21] Knapp C, Madden V, Marcu M, Wang H, Curtis C, Sloyer P, Shenkman E (2011). Information seeking behaviors of parents whose children have life-threatening illnesses. Pediatr Blood Cancer.

[22] Kishimoto K, Yoshino C, Fukushima N (2010). [Study of the health food information for cancer patients on Japanese websites]. Yakugaku Zasshi.

